# Biomechanical comparison of posterior intermediate screw fixation techniques with hybrid monoaxial and polyaxial pedicle screws in the treatment of thoracolumbar burst fracture: a finite element study

**DOI:** 10.1186/s13018-019-1149-2

**Published:** 2019-05-08

**Authors:** Huan Liu, Hongwei Wang, Jun Liu, Changqing Li, Yue Zhou, Liangbi Xiang

**Affiliations:** 1 Department of Orthopedics, Affiliated Traditional Chinese Medicine Hospital, Southwest Medical University, Luzhou, 646000 China; 2 Department of Orthopedics, General Hospital of Northern Theater Command of Chinese PLA, Shenyang, 110016 Liaoning China; 3 State Key Laboratory of Robotics, Shenyang Institute of Automation, Chinese Academy of Science, Shenyang, 110016 Liaoning China; 4 State Key Laboratory of Materials Processing and Die & Mould Technology, Huazhong University of Science and Technology, Wuhan, 430074 Hubei China; 5 State Key Laboratory of Trauma, Burn and Combined Injury, The Third Military Medical University, Chongqing, 400038 China; 6 Department of Orthopedics, Xinqiao Hospital, The Third Military Medical University, Chongqing, 400037 China

**Keywords:** Biomechanics, Thoracolumbar fracture, Hybrid, Monoaxial pedicle screw, Polyaxial pedicle screw

## Abstract

**Background:**

To compare the biomechanical characteristics of different posterior intermediate screw fixation techniques (ISFTs) with hybrid monoaxial pedicle screws (Mps) and polyaxial pedicle screws (Pps) used in thoracolumbar burst fractures.

**Methods:**

Fixation techniques are compared with regard to the von Mises stress (VMS) of the instrumentations and intradiscal pressures (IDPs) of the adjacent segments by finite element method (FEM).

**Results:**

The redistributed ROM of the fixation models with Pps fixed at the lowest segment was twice of the other fixation models in flexion and extension. The largest value of maximal VMS of a pedicle screw was located at the lowest pedicle screws when Mps are fixed at the lowest segment. The largest value of maximal VMS of the rods was decreased when more Pps are fixed at the models. Maximal IDPs of the upper adjacent segments were all larger than those of the lower adjacent segments. The maximal IDPs of the fixation model with MPs fixed at the lowest segment were larger than the other fixation models in flexion and extension.

**Conclusions:**

Polyaxial pedicle screws could be placed at the upper or the median segment for the facilitated efficient application of the connecting rod. We should focus on the adjacent segmental degeneration especially the upper adjacent segment in the fixation model with Mps fixed at the lowest segment.

## Introduction

Posterior short-segment pedicle screw fixation is widely used for the management of traumatic thoracolumbar burst fractures [1–3], posterior intermediate screw fixation technique (ISFT) at the fracture level can help improve and maintain the kyphosis correction, and the biomechanical stability also can be increased [4–18]. As a result, improved design and implantation techniques of pedicle screws such as polyaxial pedicle screws have reduced the rate of a pedicle screw and rod breakage and facilitated efficient application of the connecting rod without undue stress on the construct [5, 11, 12, 19–21]. If the heads of the pedicle screws are not in a straight line, polyaxial pedicle screws should be placed for the facilitated efficient application of the connecting rod. Compared to a monoaxial screw design, the compression and bending strength at the polyaxial head was reduced because of its own specific structural design [20, 21], but no studies have compared the hybrid monoaxial pedicle screw (Mps) and polyaxial pedicle screw (Pps) fixation techniques with regard to the range of motion (ROM), von Mises stress (VMS) of the instrumentations, and intradiscal pressures (IDPs) of the adjacent segments.

The focus of our research is to find how to provide sufficient biomechanical stability with hybrid Mps and Pps (how many Pps and location of the Pps to put) on the premise of ensuring convenient placement of rods. In the current study, biomechanical characteristics of fixation techniques including MMM (6 Mps fixated at three levels), PPP (6 Pps fixated at three levels), PMM (2 Pps fixated at upper level, 4 Mps fixated at lower two levels), MPM (4 Mps fixated at upper and lower two levels, 2 Pps fixated at median level), MMP(4 Mps fixated at upper two levels, 2 Pps fixated at lower level), MPP(2 Mps fixated at upper levels, 4 Pps fixated at lower two levels), PMP (2 Mps fixated at median level, 4 Pps fixated at upper and lower levels), and PPM (4 Pps fixated at upper two levels, 2 Mps fixated at lower level) were compared using finite element methods, redistributed ROM, VMS of instrumentations, and IDPs of the adjacent segment under displacement loading which was evaluated.

## Materials and methods

### Finite element model (FEM) and assessment indexes

**Fig. 1:**
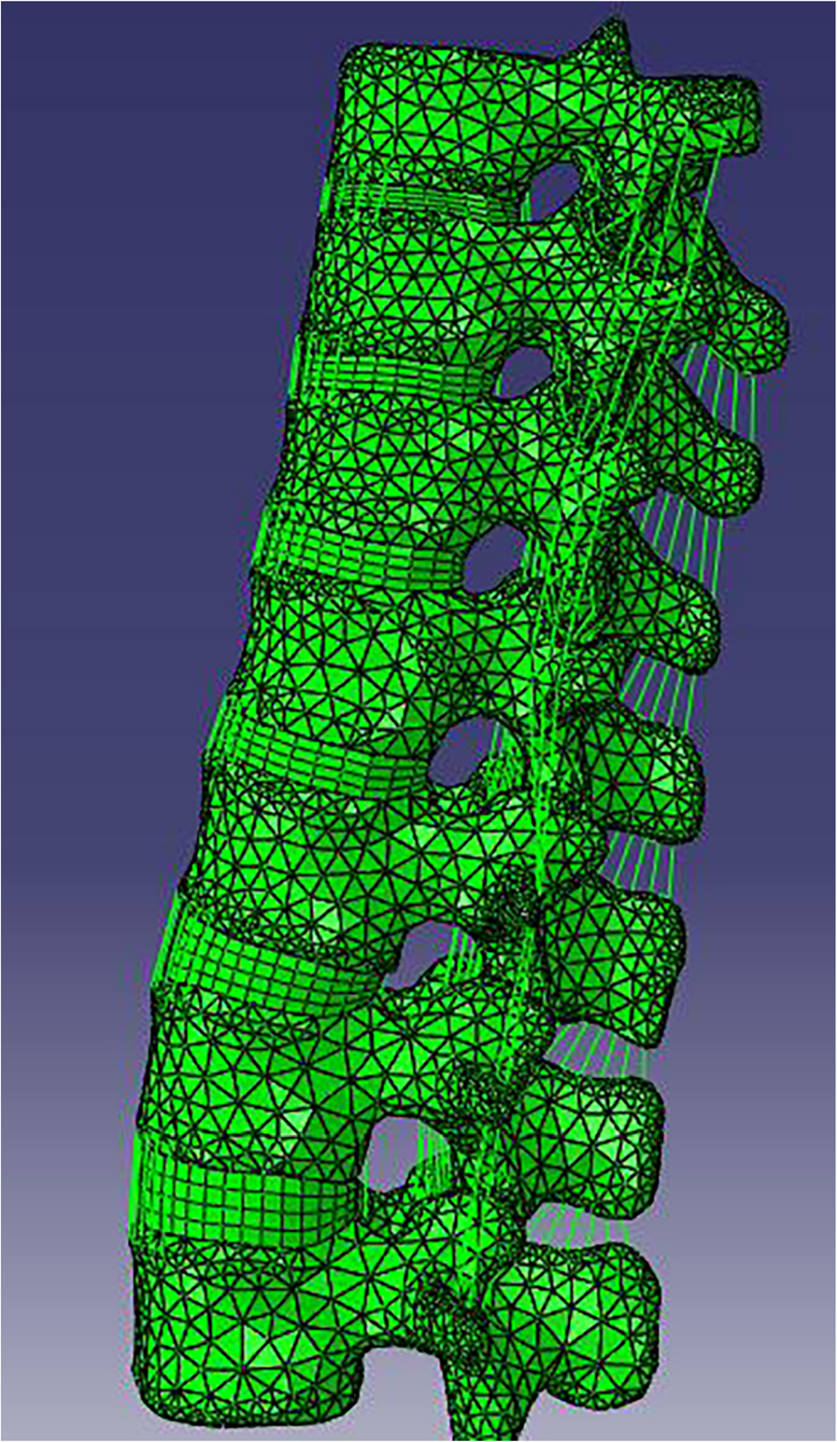
Finite element model: intact spine model. The intact spine model had a total of 20,924 nodes and 72,055 elements

A finite element model including 7 vertebrae and 6 discs between T9 and L3 of the spine obtained from 64 spiral computed tomography (CT) images of a 40-year-old healthy male (65 kg and 175 cm) without a history of spinal injury, osteoporosis, and radiographic evidence of degeneration was reconstructed and analyzed using finite element analysis software [6, 22, 23]. The CT images were scanned and imported into Mimics 10.0 (Materialise, Belgium). The surface model was then exported into Rapidform 2006 (INUS, Korea) to generate and enhance the quality of the solid model. Eventually, the model was imported into Abaqus 6.9 (Simulia) for meshing. Each vertebral body consisted of cortical bone and cancellous bone, and each vertebral disc was composed of nucleus pulposus, annulus fibrosus, and endplates. Posterior elements were built separately from the vertebral bodies. Based on a Boolean operation, the lower half of the T12 segment was resected, and the structure of the posterior part was reserved to establish a finite element model of an unstable thoracolumbar fracture. Surface-to-surface contact was defined between articulation facets. We have built the intact normal spine model and fractured spine model. The intact spine model without implants had a total of 20,924 nodes and 72,055 elements which including 48,099 tetrahedron elements, 5212 hexahedral elements, 1236 spar elements, and 17,508 shell elements (Fig. 1). We have used a truss element to replace the ligament, and the thickness of the shell element was 0.4 mm.

**Fig. 2:**
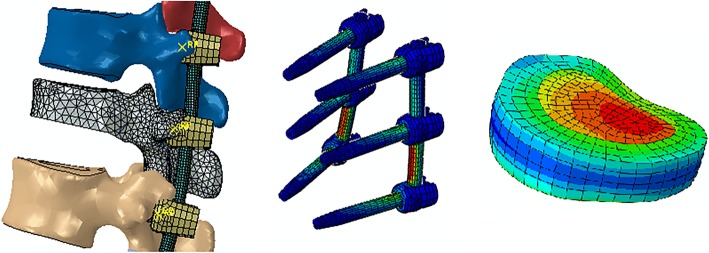
Finite element models: fracture and fixation model. Graphical figures showing the von Mises stress of pedicle screw and disc models

**Fig. 3:**
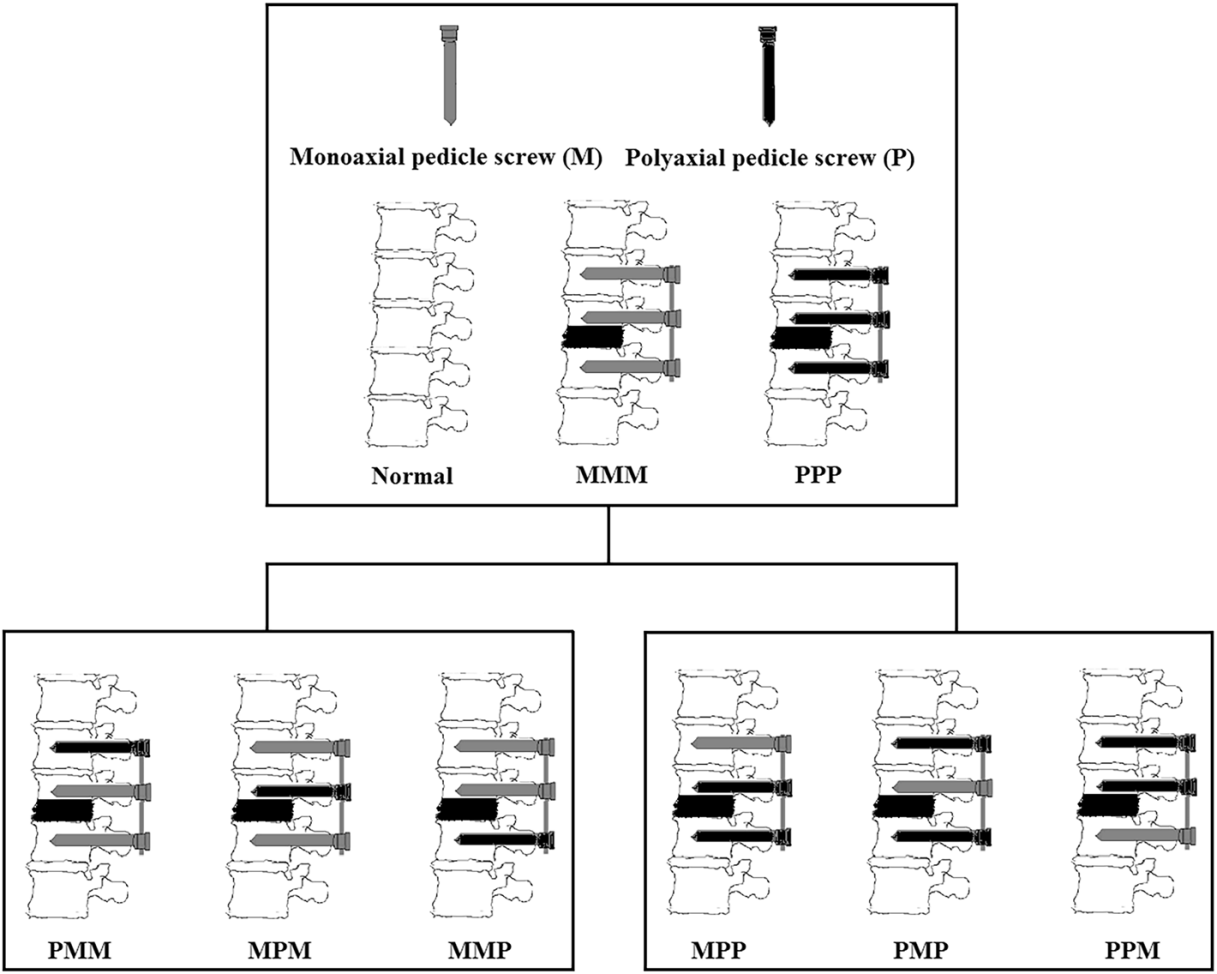
Experiment grouping design

This was a prospective study to assess the biomechanical characteristics of different posterior intermediate screw fixation techniques with hybrid Mps and Pps used in thoracolumbar burst fracture model. Fixation models were described as MMM, PPP, PMM, MPM, MMP, MPP, PMP, and PPM (Figs. 2 and 3) which may be used in the clinical practice. Surface-to-surface contact was defined between articulation facets. The element types, material properties, ligamentary cross-sectional area, and implants are shown in our previous study [6].

**Fig. 4:**
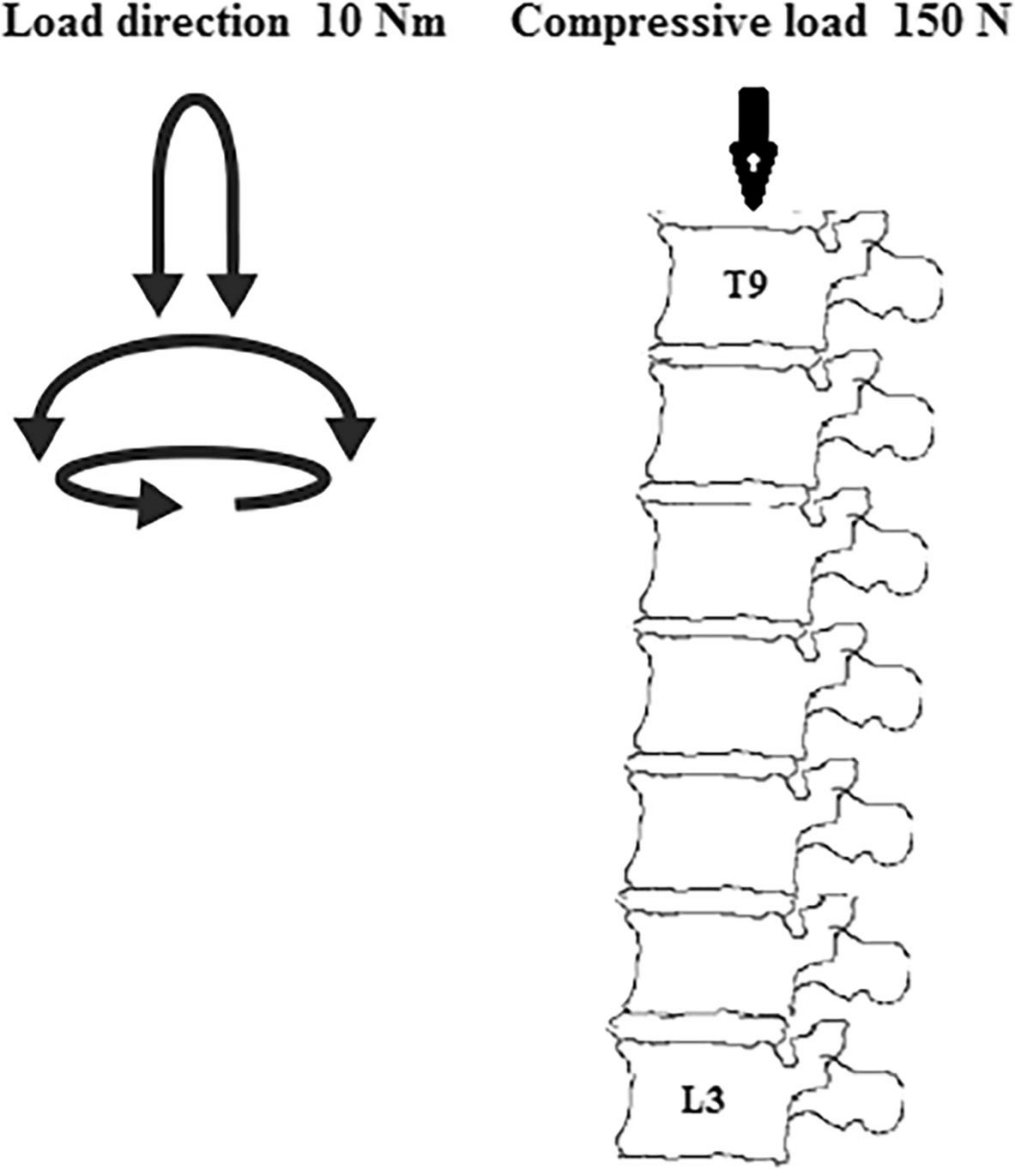
Schematic figure to show the model, boundary conditions, and applied loads

The screw diameter was 6 mm, and the screw length was 45 mm. The pedicle screws in the current study included Mps and Pps. The constraint was defined between polyaxial pedicle screw heads and shafts. However, a load limitation was defined. Surface-to-surface contact was defined between polyaxial pedicle screw heads and shafts. The screw tilt (the maximal deviation of the long axis of the screw away from perpendicular to the longitudinal rod) was 25°, the static torque was 8 Nm which meant that the polyaxial pedicle screw heads will move relative to the shafts when the torque between the heads and shafts reached 8 Nm. These parameters are referred to as the polyaxial pedicle screw of Sofamor. The top surface of T9 was applied by a pure moment of 10 Nm combined with a pre-compressive load of 150 N, the inferior endplate of L3 was constrained in all degrees of freedom (Fig. 4). To validate the rationalities of the models, including model simplification, material properties, boundary conditions, and loads, a moment of 10 Nm and a compressive load of 150 N were applied to the reference point. The range of motion (ROM) among different models was compared in our previous study [6]. There is little difference between the models. Therefore, the models in the present study are effective for further analyses.

We measured the ROM of the intact spine model T9–L3 under flexion, extension, left/right lateral bending, and left/right axial rotation and then applied ROM displacement loading to the four fixation models. The redistributed ROM of the T11–L1 segment, the largest maximal VMS of the pedicle screws and rods, and IDPs of the adjacent segment under displacement loading were evaluated. The procedure was approved by the ethics committee of Xinqiao Hospital, and the patients provided written informed consent to participate in this study.

### Statistical analysis

We used SPSS 15.0 software (SPSS Inc., Illinois, USA) to perform all statistical analyses, and *P* < 0.05 was considered significant (two-tailed). The independent sample *t* test was used to compare the means.

## Results

### ROM of the FEMs

**Fig. 5:**
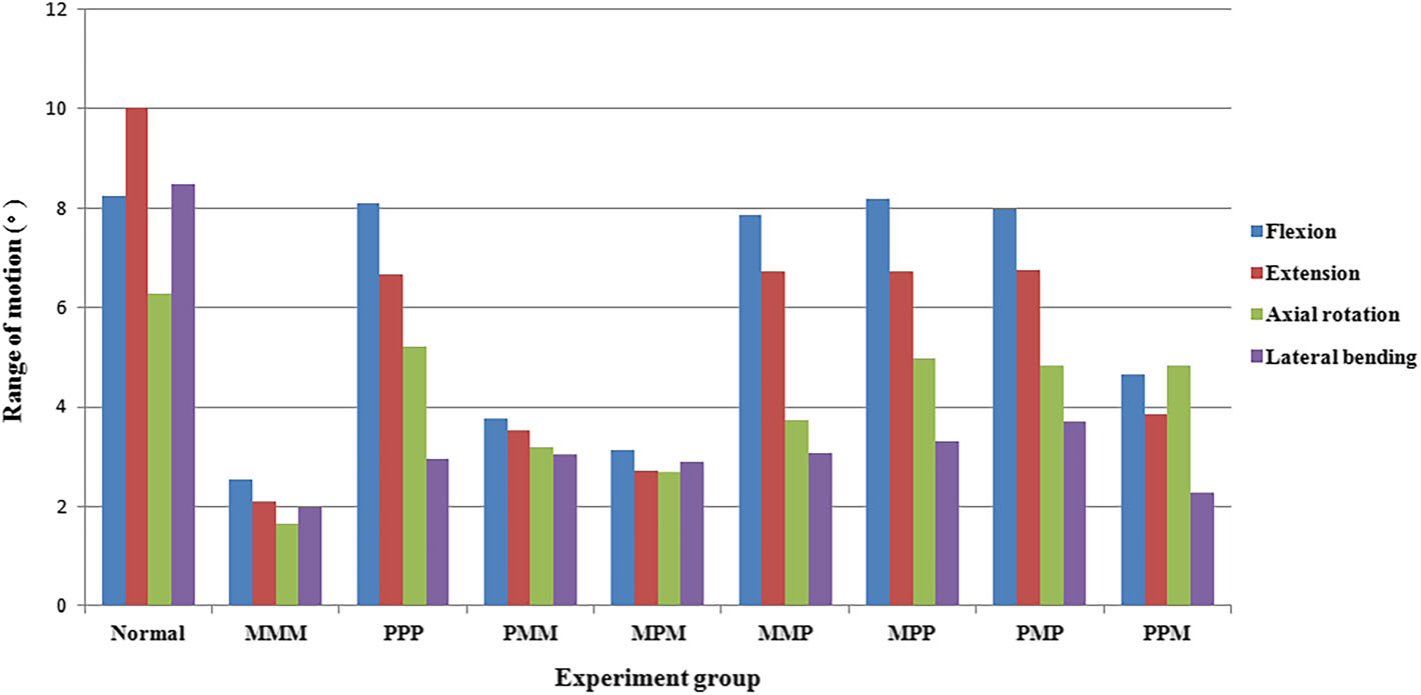
ROM of different experiment groups under different states of motion

The fixation models presented with a decreased ROM than the intact normal spine model (Table 1). The redistributed ROM of the MMM model in flexion, extension, and axial rotation was the smallest. The redistributed ROM of the fixation models with Pps fixed at the lowest segment was twice of the other fixation models in flexion and extension (Fig. 5). There were significant differences between the fixation models with Pps fixed at the lowest segment or not in the flexion (8.0 ± 0.1°, 3.5 ± 0.9°, *P* = 0.002) and extension (6.7 ± 0.1°, 3.1 ± 0.8°, *P* = 0.003), no significant differences in the axial rotation (4.7 ± 0.7°, 3.1 ± 1.3°, *P* = 0.073) and lateral bending (3.3 ± 0.3°, 2.6 ± 0.5°, *P* = 0.058).

**Table 1:** The ROM of the FEMs (°)

Variable	Normal	MMM	PPP	PMM	MPM	MMP	MPP	PMP	PPM
Flexion	8.3	2.5	8.1	3.8	3.1	7.9	8.2	8.0	4.7
Extension	10.0	2.1	6.7	3.5	2.7	6.7	6.7	6.8	3.9
Axial rotation	6.3	1.7	5.2	3.2	2.7	3.7	5.0	4.8	4.8
Lateral bending	8.5	2.0	3.0	3.1	2.9	3.1	3.3	3.7	2.3

*MMM* 6 Mps fixated at three levels; *PPP* 6 Pps fixated at three levels; *PMM* 2 Pps fixated at upper level, 4 Mps fixated at lower two levels; *MPM* 4 Mps fixated at upper and lower two levels, 2 Pps fixated at median level; *MMP* 4 Mps fixated at upper two levels, 2 Pps fixated at lower level; *MPP* 2 Mps fixated at upper levels, 4 Pps fixated at lower two levels; *PMP* 2 Mps fixated at median level, 4 Pps fixated at upper and lower levels; *PPM* 4 Pps fixated at upper two levels, 2 Mps fixated at lower level

### VMS of the pedicle screws and rods

The largest and smallest value of maximal VMS of a pedicle screw was 382.6 MPa in the PMP model and 136.9 MPa in the PPP model, respectively (Table 2). The largest value of maximal VMS of a pedicle screw was located at the lowest pedicle screws when Mps are fixed at the lowest segment. The largest and smallest value of maximal VMS of the rod was 439.9 MPa in the MMM model and 341.7 MPa in the PPP model, respectively. The largest value of maximal VMS of the rods was decreased when more Pps are fixed at the models (Table 2), but there were no significant differences between the fixation models with two Pps fixed and models with four Pps fixed (429.2 ± 10.3, 409.8 ± 15.5, *P* = 0.145).

**Table 2:** Value, location, and state of motion of pedicle screws/value and state of motion of rods with regard to the LVMS

Variable		MMM	PPP	PMM	MPM	MMP	MPP	PMP	PPM
Pedicle screws	Value (MPa)	332.8	136.9	328.2	321.3	313.9	266.1	382.6	363.1
	Location	Lower	Upper	Lower	Lower	Median	Upper	Median	Lower
	State of motion	Flexion	Flexion	Flexion	Flexion	Rotation	Rotation	Rotation	Rotation
Rods	Value (MPa)	439.9	341.7	439	430.3	418.4	414.1	392.6	422.7
	State of motion	Flexion	Rotation	Flexion	Flexion	Rotation	Rotation	Rotation	Flexion

*MMM* 6 Mps fixated at three levels; *PPP* 6 Pps fixated at three levels; *PMM* 2 Pps fixated at upper level, 4 Mps fixated at lower two levels; *MPM* 4 Mps fixated at upper and lower two levels, 2 Pps fixated at median level; *MMP* 4 Mps fixated at upper two levels, 2 Pps fixated at lower level; *MPP* 2 Mps fixated at upper levels, 4 Pps fixated at lower two levels; *PMP* 2 Mps fixated at median level, 4 Pps fixated at upper and lower levels; *PPM* 4 Pps fixated at upper two levels, 2 Mps fixated at lower level

### IDPs of the adjacent segments

**Fig. 6:**
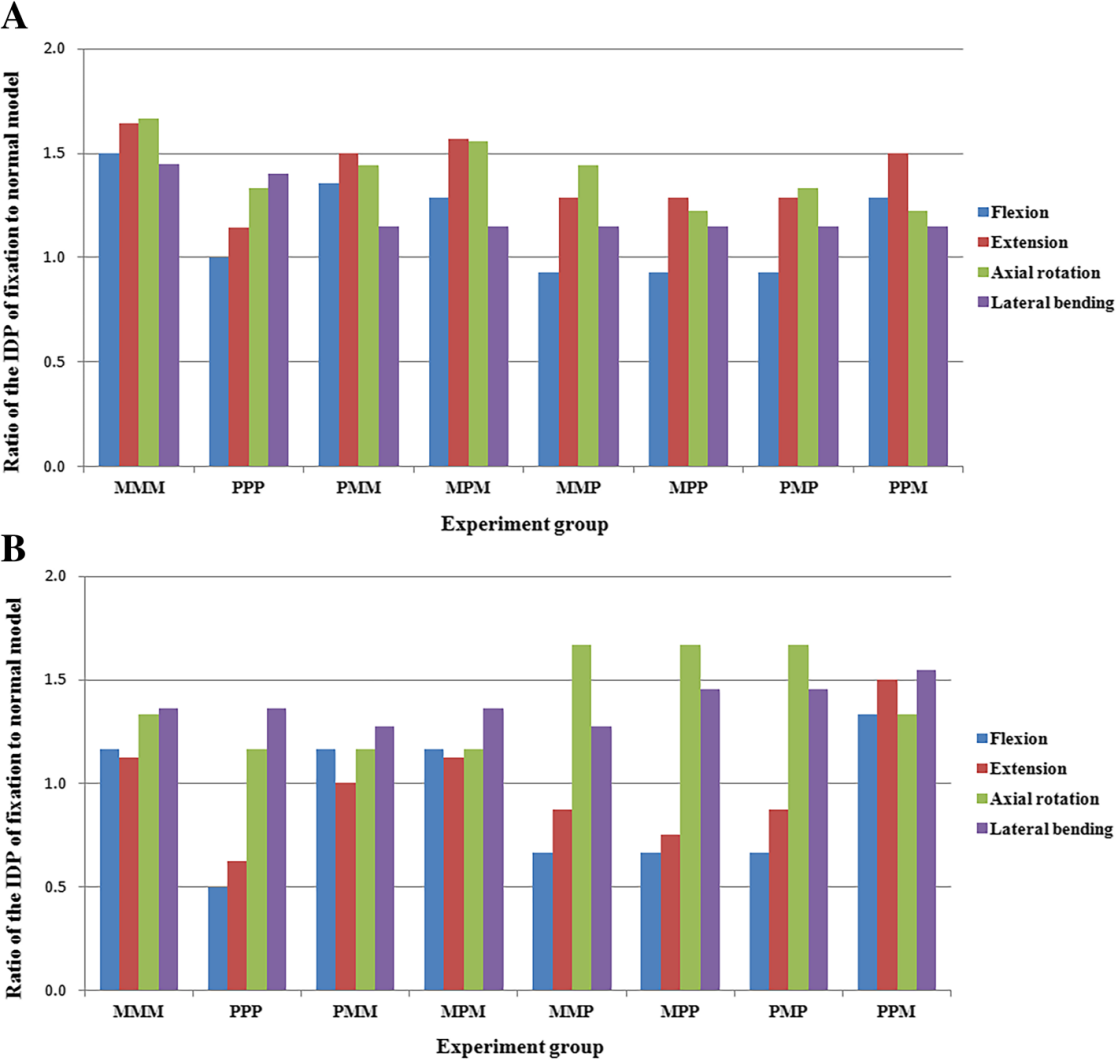
Ratio of the adjacent segmental IDPs of the fixation model to the normal model. **a** Ratio of the upper adjacent segmental IDPs of the fixation model to the normal model. **b** Ratio of the lower adjacent segmental IDPs of the fixation model to the normal model

Maximal IDPs of the adjacent segment was observed in the lateral bending. Maximal IDPs of the upper adjacent segments were all larger than those of the lower adjacent segments (Table 3). The maximal IDPs of the fixation model with Mps fixed at the lowest segment were larger than the other models in flexion and extension (Fig. 6). With regard to the upper adjacent segments, there were significant differences between the fixation models with Mps fixed at the lowest segment in the flexion (1.9 ± 0.1, 1.3 ± 0.1, *P* = 0.000) and extension (2.2 ± 0.1, 1.8 ± 0.1, *P* = 0.001), no significant differences in the axial rotation (1.3 ± 0.2, 1.2 ± 0.1, *P* = 0.235) and lateral bending (2.5 ± 0.3, 2.4 ± 0.3, *P* = 0.902). With regard to the lower adjacent segments, there were significant differences between the fixation models with Mps fixed at the lowest segment or not in the flexion (0.7 ± 0.1, 0.4 ± 0.1, *P* = 0.000) and extension (1.0 ± 0.2, 0.6 ± 0.1, *P* = 0.017), no significant differences in the axial rotation (0.8 ± 0.1, 0.9 ± 0.2, *P* = 0.072) and lateral bending (1.5 ± 0.1, 1.5 ± 0.1, *P* = 1.000).

**Table 3:** The IDPs of the upper and lower adjacent segments (MPa)

Variable	Normal			MMM			PPP			PMM			MPM			MMP			MPP			PMP			PPM	
	U	L		U	L		U	L		U	L		U	L		U	L		U	L		U	L		U	L
Flexion	1.4	0.6		2.1	0.7		1.4	0.3		1.9	0.7		1.8	0.7		1.3	0.4		1.3	0.4		1.3	0.4		1.8	0.8
Extension	1.4	0.8		2.3	0.9		1.6	0.5		2.1	0.8		2.2	0.9		1.8	0.7		1.8	0.6		1.8	0.7		2.1	1.2
Axial rotation	0.9	0.6		1.5	0.8		1.2	0.7		1.3	0.7		1.4	0.7		1.3	1.0		1.1	1.0		1.2	1.0		1.1	0.8
Lateral bending	2.0	1.1		2.9	1.5		2.8	1.5		2.3	1.4		2.3	1.5		2.3	1.4		2.3	1.6		2.3	1.6		2.3	1.7

*MMM* 6 Mps fixated at three levels; *PPP* 6 Pps fixated at three levels; *PMM* 2 Pps fixated at upper level, 4 Mps fixated at lower two levels; *MPM* 4 Mps fixated at upper and lower two levels, 2 Pps fixated at median level; *MMP* 4 Mps fixated at upper two levels, 2 Pps fixated at lower level; *MPP* 2 Mps fixated at upper levels, 4 Pps fixated at lower two levels; *PMP* 2 Mps fixated at median level, 4 Pps fixated at upper and lower levels; *PPM* 4 Pps fixated at upper two levels, 2 Mps fixated at lower level

## Discussion

Posterior intermediate screw fixation at the fracture level can help improve and maintain the kyphosis correction, and the biomechanical stability also can be increased [4–18]. However, no studies have compared the hybrid Mps and Pps fixation techniques with regard to the ROM, VMS of the instrumentations, and IDPs of the adjacent segments. Our previous study suggested that the intermediate screw fixation technique can significantly increase the stability of the spine in both the Mps fixation group and Pps fixation group. However, the Mps fixation group exhibited more stability in flexion and extension than the Pps fixation group [5]. Fixation models including MMM, PPP, PMM, MPM, MMP, MPP, PMP, and PPM showed less ROM than the intact normal spine model, and the redistributed ROM of the MMM model in flexion, extension, and axial rotation was the smallest. The redistributed ROM of the fixation models with Pps fixed at the lowest segment was twice of the other fixation models in flexion and extension. The redistributed ROMs of the PMM and MPM models were very close to the MMM model. The phenomenon can be explained for that polyaxial pedicle screw heads are vulnerable to fatigue failure; the region between the screw head and shaft was found to fail first in many biomechanical studies [19, 20, 24]. Through the study, we can see that if the heads of the pedicle screws are not in a straight line, we should place the polyaxial pedicle screws at the upper or the median segment.

The largest and smallest value of maximal VMS of a pedicle screw was 382.6 MPa in the PMP model and 136.9 MPa in the PPP model, respectively. The largest value of maximal VMS of a pedicle screw was located at the lowest pedicle screws when Mps are fixed at the lowest segment. These results may suggest that the PMP technique can increase the VMS of the pedicle screws. Upon suspecting that a pedicle screw is broken, we must focus on the median pedicle screws in PMP technique and the lower pedicle screws when Mps are fixed at the lowest segment. The largest and smallest value of maximal VMS of the rod was 439.9 MPa in the MMM model and 341.7 MPa in the PPP model, respectively. The largest value of maximal VMS of the rods was decreased when more Pps are fixed at the models. These results may suggest that the Pps technique can decrease the VMS of the rods. Upon suspecting that a rod is broken, we must focus on the MMM, PMM, and MPM fixation techniques.

In our study, the maximal IDPs of the adjacent segment were observed in the lateral bending. Maximal IDPs of the upper adjacent segments were all larger than those of the lower adjacent segments in the fixation models. These results were consistent with previous studies [25–28], the upper ASD can develop more easily than the lower ASD after the fusion surgery. The maximal IDPs of the adjacent segment in the fixation model with Mps fixed at the lowest segment were larger than the other fixation models in flexion and extension and larger than the normal model in all states of motion. These results were consistent with previous studies that noted that fusion accelerates degenerative changes at the adjacent level compared with natural history [29–31].

This study has several limitations. It is necessary to discuss several factors, including different persons, muscle force, ribs, and length and diameter of pedicle screws, for a more clinically feasible conclusion because these factors can influence finite element analysis results.

## Conclusion

ROM of the fixation models with Pps fixed at the lowest segment was twice of the other fixation models in flexion and extension, and the largest value of maximal VMS of the rods was decreased when more Pps are fixed at the models. The largest value of maximal VMS of a pedicle screw was located at the lowest pedicle screws, and the maximal adjacent segmental IDPs of the fixation model were larger than the other models in flexion and extension when Mps are fixed at the lowest segment. Through the study, we can see that if the heads of the pedicle screws are not in a straight line, the polyaxial pedicle screws should be placed at the upper or the median segment for the facilitated efficient application of the connecting rod. Upon suspecting instrumentation failure, we must focus on the median pedicle screws in the PMP fixation technique and the lower pedicle screws when Mps are fixed at the lowest segment and the rod in the MMM, PMM, and MPM fixation techniques. We should focus on the adjacent segmental degeneration especially the upper adjacent segment in the fixation model with Mps fixed at the lowest segment.

## Abbreviations

IDPs: Intradiscal pressures
ISFT: Intermediate screw fixation technique
MMM: 6 Mps fixated at three levels
MMP: 4 Mps fixated at upper two levels, 2 Pps fixated at lower level
MPM: 4 Mps fixated at upper and lower two levels, 2 Pps fixated at median level
MPP: 2 Mps fixated at upper levels, 4 Pps fixated at lower two levels
Mps: Monoaxial pedicle screws
PMM: 2 Pps fixated at upper level, 4 Mps fixated at lower two levels
PMP: 2 Mps fixated at median level, 4 Pps fixated at upper and lower levels
PPM: 4 Pps fixated at upper two levels, 2 Mps fixated at lower level
PPP: 6 Pps fixated at three levels
Pps: Polyaxial pedicle screws
ROM: Range of motion
VMS: von Mises stress
